# Network Pharmacology-Based Prediction of Active Ingredients and Potential Targets of ShengDiHuang Decoction for Treatment of Dysfunctional Uterine Bleeding

**DOI:** 10.1155/2020/7370304

**Published:** 2020-05-11

**Authors:** Hui Yang, Yu Fan, Jiangxue Cheng, Junbo Zou, Xiaofei Zhang, Yajun Shi, Dongyan Guo

**Affiliations:** ^1^Shaanxi Key Laboratory of Traditional Chinese Medicine Foundation and New Drug Research, College of Pharmacy, Shaanxi University of Chinese Medicine, Xianyang 712046, China; ^2^School of Basic Medical Science, Shaanxi University of Chinese Medicine, Xianyang 712046, China

## Abstract

**Objective:**

To analyze the potential active ingredients and related crucial targets of the ShengDiHuang Decoction (SDHD) formula in the treatment of dysfunctional uterine bleeding (DUB) by using network pharmacology and verification experiment.

**Methods:**

In this study, we determined the potential active ingredients from the traditional SDHD formula and their targets with the network pharmacology method. The network of “compound-disease-target” was constructed by the software of Cytoscape. Software of DAVID was used to enrich pathways for these 87 targets of SDHD. Further, the therapeutic effect of SDHD on DUB was verified by observing the morphological changes of the uterus and ovaries and determining the expression of ERS2 and progesterone in the plasma.

**Results:**

52 compounds of *Rheum* and 5 compounds of *Rehmannia* were selected, and 87 potential targets were screened by network pharmacology. Furthermore, 7 main active ingredients were acquired by the ADME process. In addition, enrichment analysis of drug-target networks indicated that SDHD may play a role in overall coordination through “multicomponent and multitarget” in different organ patterns by regulating multiple pathways directly or indirectly. Finally, in the verification experiment of SDHD on DUB, it was found that SDHD can effectively repair the uterus and ovaries and also have an upregulation effect on the target ESR2 and increase the content of progesterone.

**Conclusion:**

Overall, this study revealed potential mechanisms of multitarget and multicomponent about SDHD in the treatment of DUB and provided a scientific foundation for further studying the mechanism.

## 1. Introduction

Dysfunctional uterine bleeding (DUB) is defined as a state of abnormal uterine bleeding [1], which is a symptom of a serious disorder of the menstrual cycle, menstrual period, and menstrual flow [2, 3]. The broad spectrum of causes of abnormal uterine bleeding includes both genital and extra genital lesions. It can occur between menarche and menopause, at any time during the ovulation and anovulation cycles. It is known to be associated with almost any style of endometrium and ranges from normal endometrium to hyperplasia, irregular maturation, irregular exfoliation, and atrophy of chronic menstruation [4]. Among them, endometrial hyperplasia is a premalignant lesion of the uterus. A large amount of data, from the World Health Organization's multicenter survey, showed that the prevalence of menstrual bleeding ranges from 8% to 27% [5, 6]. DUB is the most common during the childbearing age. At present, administering different doses of progesterone, based on the age of person, is the most common treatment for DUB. The primary treatment for adolescents under the age of 18 is low-dose combined with hormonal contraceptive therapy (20–35 *μ*g of ethinylestradiol). For women aged 40 years or more, before menopause, the treatment includes cyclic progestin therapy, oral low-dose contraceptives, levonorgestrel intrauterine device, or cyclic hormone therapy [7]. Although these therapies are considered to have a certain effect, they have an adverse effect on the overall health of women; therefore, the development of more effective treatment strategies has important clinical implications for improving the curative treatment of patients with DUB and reducing the incidence of side effects.

Traditional Chinese Medicine (TCM) was recognized as a popular complementary and alternative medicine in Western countries [8], and it has drawn extensive attention around the world due to its satisfactory clinical efficacy. ShengDiHuang Decoction, composed of *Rehmannia* and *Rheum* (in the weight ratio of 30 : 1), is derived from the recipients of “Qianjinfang” of Sun Simiao in the Tang Dynasty [9]. It is mainly used for the treatment of hematochezia and dysfunctional uterine bleeding [10]. Modern medical research showed that *Rehmannia* has the functions of improving hematopoiesis, promoting vascular endothelial cell proliferation, and improving immunity [11, 12]. *Rehmannia*, as the principle herb, has the effect of cooling blood and hemostasis and tonifying the yin and the kidney, and *Rheum* as adjuvant herbs assist the effects of *Rehmannia* to promote blood circulation to remove blood stasis [13]. Although studies have shown that SDHD contributes to the treatment of dysfunctional uterine bleeding, the mechanism of “multicomponent and multitarget” therapeutic of this formulation remains unclear due to its complex nature.

**Figure 1:**
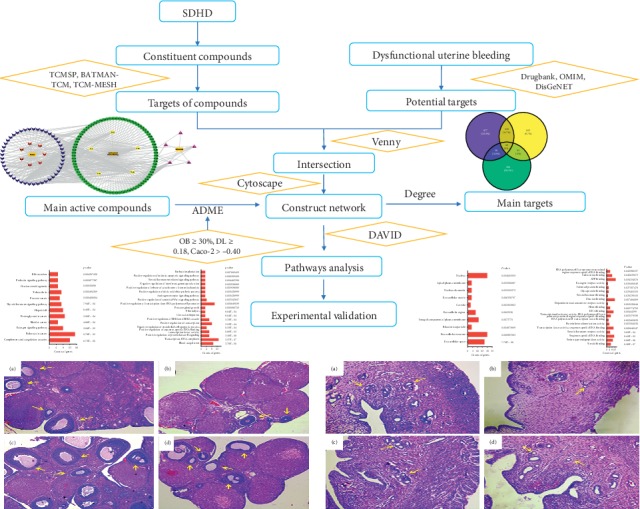
Experimental flowchart of this study based on network pharmacology and experimental validation for illustrating pharmacological mechanisms of SDHD acting on dysfunctional uterine bleeding.

Network pharmacology is a promising strategy for systemic analysis based on interaction networks of diseases-genes-targets-drugs [14, 15], while providing a possible strategy from a holistic perspective to elucidate the mechanism of the action of multicomponent drugs [16]. It was reported that network pharmacology predicts the clinical efficacy, pathways, and side effects of drugs by constructing the networks of drug-drug, disease-drug, and disease-disease, providing valuable information for improving the clinical efficacy, reducing toxicity, and elucidating multiple mechanisms of drugs [17]. Because TCM herbal formulas are considered to be multicomponent and multitarget treatment, the methodology of network pharmacology applies to the prior knowledge of pursuing the combination of rules embedded in these formulas [18]. Therefore, the application of network pharmacology in TCM provides a new opportunity to understand the interaction between active compounds and related targets, which highlights the mechanisms of action [8]. Using this background, the aim of this study was to develop a method which was based on comprehensive network pharmacology to reveal the relationship between the targets of DUB and active ingredients involved in the SDHD and to explore the potential therapeutic mechanisms of SDHD for treating DUB. The experimental flowchart of our present study is shown in Figure 1.

## 2. Materials and Methods

### 2.1. Materials

*Rehmannia* was purchased from Baoji Hanfang National Herbal Pieces Co., Ltd. *Rheum* was purchased from Lanzhou Xukang Pharmaceutical Co., Ltd. Co., Ltd. Gongxuening Capsules were provided by Yunnan Baiyao Group Co., Ltd., hydrocortisone came from Tianjin Jinyao Pharmaceutical Co., Ltd., hydroxycarbamide was from Beijing Jialin Pharmaceutical Co., Ltd., mifepristone was obtained from Zhejiang Xianyi Pharmaceutical Co., Ltd., and misoprostol was from China Resources Zizhu Pharmaceutical Co., Ltd. The ERS2 kit was purchased from Wuhan Huamei Biological Engineering Co., Ltd. The progesterone kit was supplied from the Nanjing Institute of Bioengineering.

### 2.2. Network Construction

#### 2.2.1. Collection of Component Targets

All compounds of the two Chinese medicinal herbs in SDHD were collected from the TCMSP (http://lsp.nwu.edu.cn/tcmsp.php), BATMAN-TCM (http://bionet.ncpsb.org/batman-tcm), and TCM-MESH (http://mesh.tcm.microbioinformatics.org/). Compounds were selected with DL > 0.18 in TCMSP. According to the collected compounds, the potential targets of the ingredients were searched in the database of TCMSP, BATMAN-TCM, and TCM-MESH. Other than these, we also performed the validated targets which were obtained from the HIT database (http://lifecenter.sgst.cn/hit) and the predicted targets which were acquired from the ChemMapper database (http://lilab.ecust.edu.cn/chemmapper/).

#### 2.2.2. Collection of Disease-Targets

Disease-targets were collected from the Drugbank (http://www.drugbank.ca/) and OMIM (http://www.omim.org/) with dysfunctional uterine bleeding as keywords, as well as obtained from DisGeNET (http://www.disgenet.org/web/DisGeNET/menu/home) using uterine hemorrhage as keywords.

#### 2.2.3. Network Construction

All the gene names of *Rheum* ingredient-targets, the *Rehmannia* ingredient-targets, and the disease-targets were converted to a Uniport ID in the Uniprot (https://www.uniprot.org/) and then merged and intersected by mapping the Venny (http://bioinfogp.cnb.csic.es/tools/venny/index.html). After that, 52 ingredients of *Rheum*, 5 ingredients of *Rehmannia*, and 87 related targets were collected and used as an input to the software Cytoscape (version3.2.1) to construct the predicted-target (PT) network.

### 2.3. Screening of Main Active Compounds

OB is one of the most important pharmacokinetic parameters in the ADME process [19]. High OB is usually a key index for determining the DL of the active molecule. In the early stages of drug development, DL assessment helps to screen out major compounds and increase the “hit rate” of drug candidates. The average DL value of all drugs is 0.18 in the Drugbank database, indicating a high DL value [20]. Caco-2 cells can be used to study intestinal epithelial permeability [21]. Drugs for oral administration are mainly absorbed by intestinal epithelial cells. Therefore, it is crucial for predicting drug transport across the monolayer to simulate the monolayer of the intestinal epithelial cells, while molecules with Caco-2 > −0.40 are considered to have good permeability in the intestinal epithelium. Therefore, the candidate compounds we selected need to meet the requirements of OB ≥ 30%, DL ≥ 0.18, and Caco-2 > −0.40 [20, 22].

### 2.4. Screened Hub Node

Four topological properties, “degree,” “betweenness centrality,” “closeness centrality,” and “average shortest path length” were analyzed in Cytoscape (version3.2.1) and then the degree values of which are more than two folds of the median degree of all nodes in the network were filtered, which are considered to be the important targets [23].

### 2.5. Pathway Enrichment Analysis

To clarify the pathways which are involved in putative SDHD targets, KEGG and Go enrichment analysis was performed using the Database Visualization and Integrated Discovery software32 (DAVID, http://david.abcc.ncifcrf.gov/home.jsp, version 6.8) and *p* < 0.05 was selected, and then the pathway data obtained from the Kyoto Encyclopedia of Genes and Genomes (KEGG, https://www.kegg.jp/) were selected.

### 2.6. Animal Experiment to Verify the Efficacy

Healthy Sprague Dawley (SD) rats (230 ± 20 g) were used and supplied by the Chengdu Dashuo Animal Experiment Center. Rats were raised under standard temperature, humidity, and light conditions and fed standard rodent diet and water. The study was approved by the Ethical Committee of the Shaanxi University of Chinese Medicine.

Rats were caged with female : male in the ratio of 2 : 1. The next day, the female rats were examined for vaginal suppository and vaginal smear to find whether there is sperm in the vaginal suppository and vaginal smear as the first day of pregnancy. From the first day of pregnancy, all groups were oral administered with hydroxycarbamide at a dose of 450 mg/kg at 8:00 am and injected 3.7 mg/kg of hydrocortisone every day, except for the control group. The treatment was continued for 7 days to make a model of functional dysfunctional uterine bleeding. On the 8th day of pregnancy, mifepristone and misoprostol were administered orally at 8:00 am and at 18:00, respectively. At the same time, a sterile cotton ball was placed in the vagina of each pregnant rat. The next morning, the cotton ball of the rat's vagina was collected, and blood was seen on the cotton ball, indicating that the model was successful. After successful modeling, twenty one successful model rats were randomly divided into the model group, the positive group, and the treatment group with seven rats in each group. The positive group and the treatment group were administered with 0.156 g/kg Gongxuening and 0.0646 g/kg SDHD on the second day for 8 consecutive days, respectively. After the last administration, the rats were fasted with free access to drinking water. On the next day, the rats were anesthetized and blood was taken from the abdominal aorta. Afterwards, the uterus and ovary tissues were taken and fixed in a 10% formalin solution for 24 hours and then sequentially dehydrated with an ethanol gradient, embedded in paraffin, and sliced, and then hematoxylin-eosin (HE) staining was done. The morphological and quantity changes of the uterine glands in the uterus and follicles in ovaries were observed under a microscope. In addition, the determination of ERS2 and progesterone in the serum was performed.

## 3. Results and Discussion

### 3.1. Network Construction

#### 3.1.1. Collection of Targets

A total of 65 constituent compounds of the 2 herbs contained in SDHD were collected, including 57 of *Rheum* and 8 of *Rehmannia*. 1085 proteins were predicted for *Rheum* ingredients, and 319 proteins of *Rehmannia* ingredients were fished. 415 related targets for dysfunctional uterine bleeding were collected.

#### 3.1.2. Network Construction and Analyze

**Figure 2:**
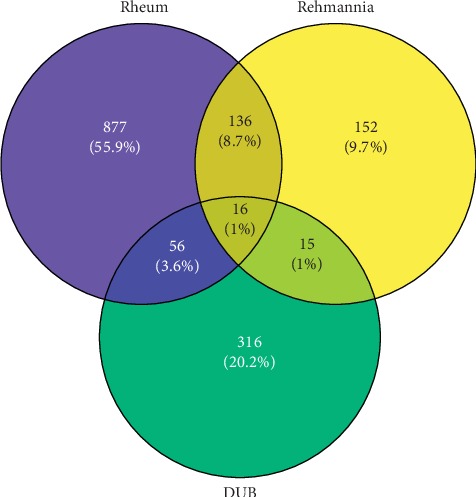
The intersection of compound-targets and disease-targets: blue represents the targets of ingredients in *Rheum*, yellow represents the targets of ingredients from *Rehmannia*, and the targets of dysfunctional uterine bleeding are represented by green.

**Figure 3:**
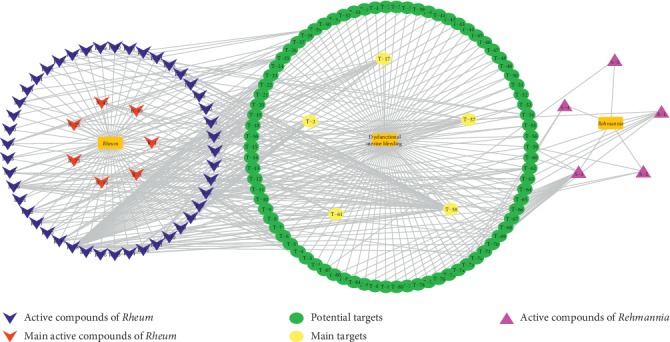
The network of “compound-disease-target.”

As shown in Figure 2, through the mapping of the Venny diagram about the compound-targets and the disease-targets, it was found that 87 targets, 52 compounds of *Rheum*, and 5 compounds of *Rehmannia* were screened. The chemical names, structures, and molecular formulas of all compounds are shown in Supplementary Table 1, and the target name and its Uniport ID are shown in Supplementary Table 2. After that, the network of “compound-disease-target” was constructed by the software Cytoscape (version 3.2.1). As shown in Figure 3, this network contains 146 nodes and 294 edges, and at the same time, we also found that each target was connected with several ingredients in the network. For example, PTGS2 was targeted by aloe emodin, aucubin, chrysophanol, emodin, and rhein; ESR1 was targeted by serotonin, rhein, emodin campesterol, and physcion; PGR was targeted by emodin anthrone, palmidin C, progesterone, and serotonin. All these results suggested that multiple targets were associated with a variety of compounds in different herbs, which might exhibit synergistic effects or additive effects of SDHD in the treatment of dysfunctional uterine bleeding.

### 3.2. Screening of Main Active Compounds

Through the screening of main active ingredients by the ADME process, we found that seven components in *Rheum* meet the requirements of OB ≥ 30%, DL ≥ 0.18, and Caco-2 > −0.40, including aloe emodin (anthraquinone), (−)-catechin (phenols), beta-sitosterol (triterpene), daucosterol (triterpene), eupatin (flavonoid), rhein (anthraquinone), and toralactone (phenols). Although no ingredients in *Rehmannia* meet the requirements, catapol as the highest content compound in *Rehmannia* can be considered as the main active ingredient [24]. The values of OB, Caco-2, and DL of the main active compounds are shown in Table 1.

### 3.3. Screened Hub Node

Predicted SDHD target-known therapeutic targets of the dysfunctional uterine bleeding network were constructed using the links between targets of SDHD and known therapeutic targets of disease. As previously reported [23], if the values of the degree are greater than twice the median degree of all the nodes in a network, the node can be used as a hub. Moreover, four topological properties, “degree,” “betweenness centrality,” “closeness centrality,” and “average shortest path length,” were calculated to screen the targets for topological importance. The definitions of the four topological features listed earlier are provided in Supplementary Table 3. It was showed the degree of PTGS2, ESR1, PGR, ESR2, and F10 is 35, 15, 12, 11, and 10, respectively, which were considered as important targets.

PTGS2 expresses cyclooxygenase (COX-2), and it was reported that cyclooxygenase is the main rate-limiting enzyme in the synthesis of prostaglandins and thromboxane from arachidonic acid, COX-1 is a structural enzyme, and COX-2 is an induced enzyme [25]. Symptoms of abnormal uterine bleeding and dysmenorrhea are associated with increased prostaglandin levels, while COX-2 was associated with angiogenic markers *in vitro* [26]. Under normal physiological conditions, the expression level of COX-2 is extremely low, and overexpression of COX-2 in vascular smooth muscle cells leads to an inflammatory reaction of the vessel wall, instability of the plaque, and intimal hyperplasia [27]. Current research revealed that Chinese medicine promoting blood circulation and blood-breaking can significantly downregulate the expression level of PTGS2 [25]. Therefore, it may lead to a decrease in the expression of COX-2 and improve the fluidity of blood. ESR1, estrogen receptor-*α*, plays an important role in female reproductive activity. Related studies have shown that deregulation of estrogen expression may lead to changes in endometrial function [28, 29]. The endometrium undergoes extensive vascular growth, remodeling, and regression approximately once a month, and those are primarily regulated by the ovarian steroid hormones estrogen and progesterone [30]. Abnormal expression of estrogen may cause abnormal angiogenesis and further lead to several different endometrial-related pathologies, including endometrial cancer, endometriosis, menstruation, and breakthrough bleeding [31]. ESR2, estrogen receptor-*β*, is a nuclear hormone receptor and binds estrogens with an affinity similar to that of ESR1 and activates expression of reporter genes containing estrogen response elements (ERE) in an estrogen-dependent manner. It has been well determined that estrogen is involved in the pathophysiology of breast cancer. Estrogen receptor-*α* can promote the proliferation of cancer tissues, while ER-*β* can protect against the mitogenic effect of estrogen in breast tissue. The expression of ER-*α* and ER-*β* may greatly influence the development, treatment, and prognosis of breast cancer [32]. Previous studies using knockout mice of ER-*β* have shown that deficiency of ER-*β* leads to hyperproliferation and loss of differentiation of the uterus [33], ventral prostate [34], and colon in epithelia [35]. Thence, it is speculated that SDHD may avoid the change of endometrial function by regulating the expression of estrogen. PGR, progesterone receptor, is an intracellular protein that is activated by the steroid hormone progesterone. Progesterone, acting through the PGR, is one of the most critical regulators of endometrial differentiation and helps regulate the cyclic changes in endometrial tissue [36, 37]. Related studies have found the gene network that operates downstream of each PGR isoform, which contains PGR-A and PGR-B, to mediate critical functions of regulation of the cell cycle and angiogenesis [37]. Uterine has been shown to contain and produce angiogenesis and antiangiogenesis factors [38]. Angiogenesis-related genes found that downstream of PGR-B included vascular endothelial growth factor A, angiopoietin 2, angiopoietin-like 4, and fibroblast growth factor 2. Therefore, SDHD may affect angiogenesis-related genes of uterine by regulating PGR and further affect the treatment of DUB. F10, Factor X, is a vitamin K-dependent glycoprotein and also a pivotal coagulation factor in the common coagulation pathway that converts prothrombin to thrombin in the presence of factor Va, calcium, and phospholipid during blood clotting [39]. Factor X deficiency (FXD) is a rare coagulation with manifestation of variable severity and usually increase the risk of bleeding complications [40]. It is reported that heavy menstrual bleeding (HMB), recurrent ovulation bleeding with hemoperitoneum, and bleeding complications in pregnancy are more common in women [41]. At the same time, it was also found that combined coagulation factor VII (FVII) and factor X (FX) deficiency (combined FVII/FX deficiency) belongs to the group of bleeding disorders in which both factors show reduced plasma activity [42]. So, it is speculated that SDHD may reduce uterine bleeding by regulating the expression of F10.

### 3.4. Pathway Enrichment Analysis

**Figure 4:**
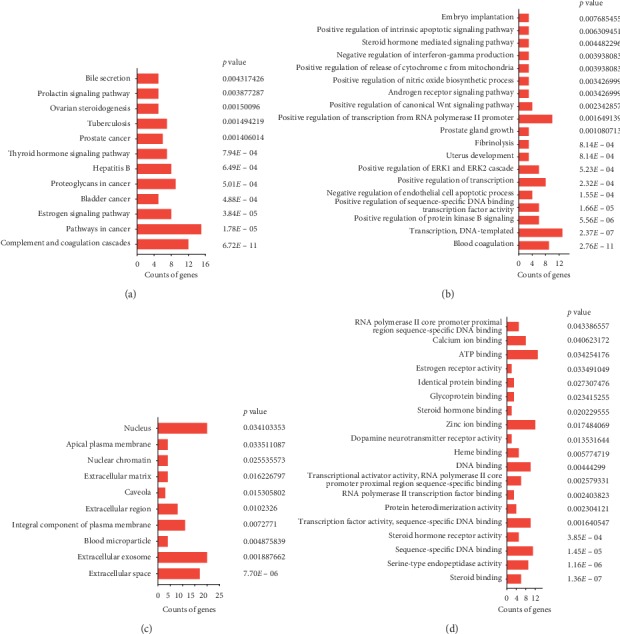
GO functional analysis by DAVID of SDHD on DUB. (a) KEGG pathways, (b) biological processes terms, (c) cell component terms, and (d) molecular function terms. The *x*-axis is the count of genes for different pathways, and the *y*-axis shows the different pathways to treat DUB.

In order to understand the mechanism of SDHD on treating DUB and to identify signaling pathways associated with SDHD protein targets, DAVID software was used to enrich pathways for these 87 targets of SDHD. A total of 30 pathways of KEGG were obtained, and the results of all pathways are shown in Supplementary Table 4, which includes the name of pathways, the genes, the count, the *p* value, and the FDR. As shown in Figure 4(a), 12 pathways with a *p* value less than 0.05 were possible pathways for SDHD treatment of DUB. The results indicated that these proteins are closely related to the immune system, endocrine system, and cancer, such as complement and coagulation cascades, pathways in cancer, estrogen signaling pathway, bladder cancer, proteoglycans in cancer, and hepatitis B. GO analysis involved three main terms, including biological processes (BP), cellular components (CC), and molecular functions (MF). For GO-BP, 89 pathways were obtained (Supplementary Table 5), including 19 pathways within a *p* value 0.01, and these pathways are shown in Figure 4(b). In addition, there are 14 GO-CC pathways, shown in Supplementary Table 6, of which 10 pathways, shown in Figure 4(c), with a *p* value less than 0.05 are considered more likely to be involved in the treatment of DUB. As shown in Supplementary Table 7, 23 GO-MF pathways were obtained in the pathway enrichment. Also, 19 pathways have a *p* value of less than 0.05, which is shown in Figure 4(d). PTGS2, ESR1, PGR, ESR2, and F10, which were considered as important targets, played a significant role in the different pathways. Then, we choose the top three pathways of KEGG for analysis.

The pathway of complement and coagulation cascades is a proteolytic cascade in blood plasma and a mediator of innate immunity, a nonspecific defense mechanism against pathogens. There are three pathways of complement activation: the classical pathway, the lectin pathway, and the alternative pathway. All these pathways generate a crucial enzymatic activity, which in turn produces the effector molecules of complement. The main consequences of complement activation are the opsonization of pathogens, the recruitment of inflammatory and immunocompetent cells, and the direct killing of pathogens. Blood coagulation is another series of proenzyme-to-serine protease conversion, culminating the formation of thrombin, the enzyme responsible for the conversion of the soluble fibrinogen to the insoluble fibrin clot. The coagulation system has evolved to limit blood loss at sites of vascular injury, and its biological effects are usually mediated directly by enzymes. In the case of vascular injury, thrombin is produced very rapidly and relatively in large quantities at the site of injury. Therefore, effective inhibitors are needed to ensure that excessive coagulation does not occur [43]. It was reported that gamma-aminobutyric acid, from *Rehmannia*, participates in the activation of thrombus, which significantly prolongs the closure time of whole blood and the occlusion time of platelet embolism, and plays an important role in hemostasis [44]. Acteoside, from *Rehmannia*, has antiplatelet aggregation effect, thereby inhibiting blood coagulation [45]. The above results suggested that gamma-aminobutyric acid and acteoside may cure uterine bleeding by adjusting the pathway of complement and coagulation cascades. The uterine endometrial carcinoma is one of the most common malignancies in the female genital tract, and the pathways in cancer are involved in its occurrence. Approximately, 80% of endometrial carcinoma occur in menopausal or postmenopausal women and the remaining 20% in premenopausal women. Abnormal genital bleeding is the most important symptom of endometrial carcinoma. Over 90% of patients appeared with abnormal bleeding, including patients with early-stage tumors. Endometrial hyperplasia and endometrial cancer usually express PR, especially well-differentiated endometrioid carcinoma, but their growth is inhibited by progesterone [46]. Therefore, progesterone, which is from *Rheum*, may play a key role in the regulation of pathways in cancer. In the estrogen signaling pathway, estrogen, as a main steroid sex hormone, can regulate a plethora of physiological processes in mammals, including the growth, development, and function of the female reproductive system, bone integrity, cellular homeostasis, and behavior. There is also substantial evidence for an effect of estrogen on the cardiovascular system [47]. A study has examined the amount of ER-*α* and ER-*β* in coronary arteries of premenopausal and postmenopausal women. It was found that increased expression of ER-*β* is associated with advanced atherosclerosis and calcification regardless of age or hormone status [48]. Furthermore, in mice with genetic ER-*α* deletion, the cardioprotective role of estrogen in ischemia/reperfusion injury is lost [49]. Thus, the vasoprotective effects of estrogen may be largely mediated by ER-*α* [50].

### 3.5. Animal Experiment to Verify the Efficacy

**Figure 5:**
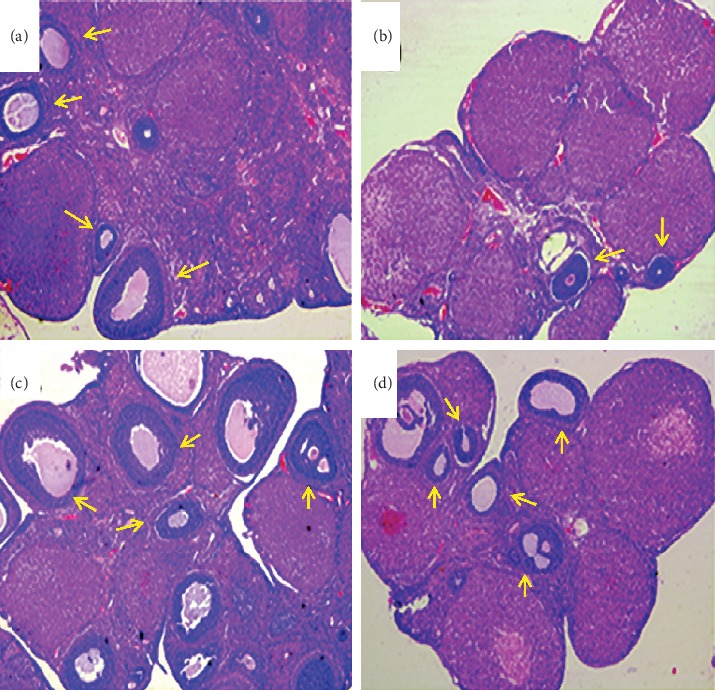
The effects of SDHD on morphological changes of ovary (HE, ×100). (a) Control group, (b) model group, (c) positive group, and (d) SDHD group.

The structure of rat ovary was observed by HE staining. In Figure 5, the number of primordial follicles in the ovarian cortex of the control group is shown. The number of follicles in the developmental stage was more, the secondary follicles were mostly in the superficial cortex, and the follicular cavity was obvious. In the model group, the number of follicles was significantly reduced, and the interstitial connective tissue number increased. There was a significant difference compared with the control group. The number of follicles in the ovarian development group was higher in the positive group and the SDHD group, and more secondary follicles were more common. Compared with the model group, the difference was significant.

**Figure 6:**
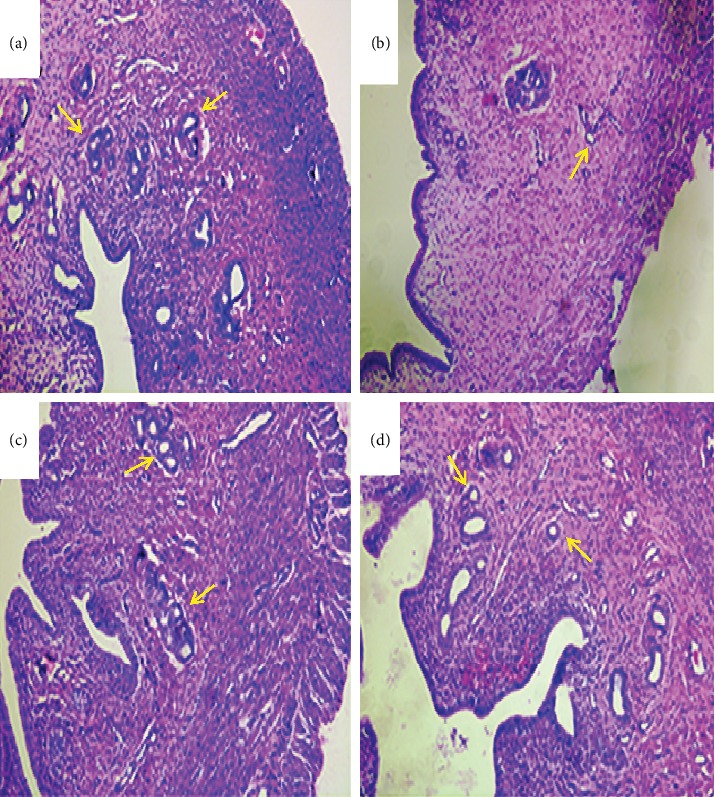
The effects of SDHD on morphological changes of uterus (HE, ×100). (a) Control group, (b) model group, (c) positive group, and (d) SDHD group.

The morphology changes of uterus are shown in Figure 6. In the control group, the endometrium was thicker and the uterus glandular cavity was obvious. Compared with the control group, the model group had a thinner endometrium, smaller glandular cavity, and smaller stromal cell, as well as fewer uterine glands. In the positive group, the endometrium was thick, and the number of uterine glands is large and the secretions are visible. The positive group and the SDHD group have thicker endometrium, more glands, and stromal cell hypertrophy than the model group. These results suggested that SDHD has a certain repairing effect on the uterus and ovaries of DUB.

**Figure 7:**
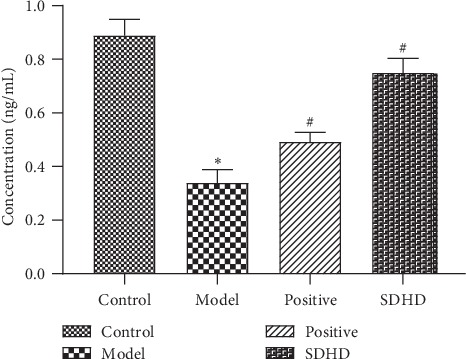
Effect of SDHD on ESR2 of DUB. ^*∗*^*p* < 0.05, compared with the control group. ^#^*p* < 0.05, compared with the model group.

ESR2, the nuclear hormone receptor, has well determined that estrogen is involved in the pathophysiology of breast cancer. Deficiency of ER-*β* leads to hyperproliferation and loss of differentiation of the uterus [33], ventral prostate [34], and colon in the epithelia [35]. It was involved in the estrogen signaling pathway and the prolactin signaling pathway in the KEGG pathway and participated in transcription, DNA-templated, positive regulation of sequence-specific DNA binding transcription factor activity and positive regulation of transcription, and DNA-templated of GO-BP and played a part in the nucleus of GO-CC, as well as involved in steroid binding, sequence-specific DNA binding, transcription factor activity, zinc ion binding, and estrogen receptor activity of GO-MF. So, the effect of SDHD on ESR2 of DUB was studied. As shown in Figure 7, when compared with the blank group, the ESR2 in the model group was significantly decreased, and the ESR2 in the positive group and the SDHD group was significantly higher than that in the model group. It was suggested that SDHD can indeed improve the decrease of ESR2 and further affect the uterine and ovarian damage caused by DUB.

**Figure 8:**
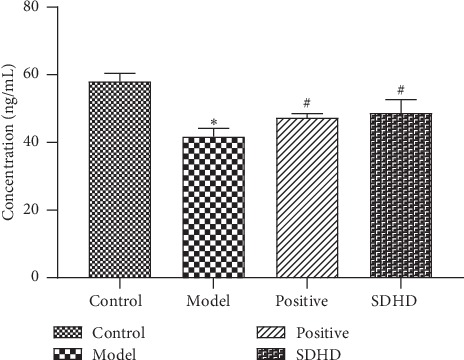
Effect of SDHD on progesterone of DUB. ^*∗*^*p* < 0.05, compared with the control group. ^#^*p* < 0.05, compared with the model group.

Progesterone, a female sex hormone produced by the corpus luteum in early pregnancy, induces secretory changes in the uterine lining [51]. It was also a natural progesterone secreted by the ovary and plays a major role in maintaining pregnancy. The content of the progesterone is an important indicator for evaluating the function of the ovary [52]. Therefore, the progesterone content in the serum was measured. As shown in Figure 8, it was found that the content of progesterone was significantly reduced in the model group, which was compared with the control group. After treatment with SDHD, the content of progesterone was significantly higher than that of the model group. It was indicated that SDHD can increase the progesterone and then acts through the PGR to regulate changes in endometrial tissue [36]. In the screening of network pharmacology pathways, we found that PGR is mostly involved in molecular function in GO analysis, such as steroid binding, sequence-specific DNA binding, steroid hormone receptor activity, transcription factor activity, transcriptional activator activity, RNA polymerase II core promoter proximal region sequence-specific binding, and Zinc ion binding.

At present, we screened SDHD for DUB targets through network pharmacology and combined with experiments, it was found that SDHD further affects the uterine and ovarian damage caused by DUB by improving the reduction of ESR2 and regulates the changes of endometrial tissue by increasing progesterone level. This method and results provided the basis for further mechanism research on SDHD for DUB treatment and also supplied a novel strategy for exploring the molecular mechanisms of traditional medicines. However, since the compound-targets of *Rehmannia* is less studied, there may be a lack in data collection, and at the same time, the mechanism of action of some targets is still unclear due to less experimental verification. Therefore, we will use more systematic methods in subsequent experiments.

## 4. Conclusion

In this study, the protein targets of DUB and active components of SDHD were collected and then the complex network between drugs and diseases were revealed by using network pharmacology. We found that the pharmacological activities of SDHD on DUB might be associated with the regulation of five main targets that include PTGS2, ESR1, PGR, ESR2, and F10. Also, SDHD has an upregulation effect on target ESR2 and increases the content of progesterone that we have confirmed through experimental verification. However, further experimental validations of these prediction results are required in our future studies.

## Figures and Tables

**Table 1:** The values of OB, Caco-2, and DL of the main active compounds.

ID	Chemical name	Structure	OB	Caco-2	DL
R-1	(−)-Catechin	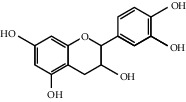	49.6764	−0.0275	0.2416
R-3	Aloe emodin	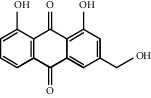	83.3796	−0.1170	0.2409
R-13	Eupatin	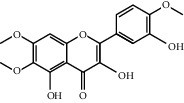	50.8031	0.5325	0.4080
R-23	Rhein	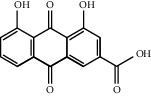	47.0652	−0.1989	0.2768
R-25	Toralactone	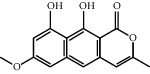	46.4636	0.8552	0.2397
R-43	Beta-sitosterol	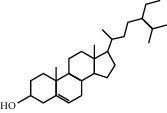	36.9139	1.3246	0.7512
R-49	Daucosterol_qt	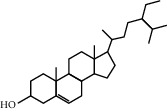	35.8889	1.3507	0.7042

## Data Availability

The data used to support the findings of this study are included within the article and the supplementary materials.

## Supplementary Materials

Supplementary Materials “Supplementary Table 1: the information of active compounds. Supplementary Table 2: the code and Uniprot ID of potential targets. Supplementary Table 3: the topological properties of the potential targets. Supplementary Table 4: the KEEG for the treatment of DUB by SDHD. Supplementary Table 5: the GO-BP pathways for the treatment of DUB by SDHD. Supplementary Table 6: the GO-CC pathways for the treatment of DUB by SDHD. Supplementary Table 7: the GO-MF pathways for the treatment of DUB by SDHD.”
