# Supplementary material for: Network Pharmacology-Based Prediction of Active Ingredients and Potential Targets of ShengDiHuang Decoction for Treatment of Dysfunctional Uterine Bleeding
Source: Evid Based Complement Alternat Med. 2020 May 11;2020:7370304. doi: 10.1155/2020/7370304 (PMC7240676; doi:10.1155/2020/7370304)
Supplement: Supplementary Materials — “Supplementary Table 1: the information of active compounds. Supplementary Table 2: the code and Uniprot ID of potential targets. Supplementary Table 3: the topological properties of the potential targets. Supplementary Table 4: the KEEG for the treatment of DUB by SDHD. Supplementary Table 5: the GO-BP pathways for the treatment of DUB by SDHD. Supplementary Table 6: the GO-CC pathways for the treatment of DUB by SDHD. Supplementary Table 7: the GO-MF pathways for the treatment of DUB by SDHD.” [file 7370304.f1.pdf]

Supplementary Table 1 The information of active compounds

ID	Chemical name	Formula	Structure	Herb name
R-1	(-)-Catechin	$C_{15}H_{14}O_6$	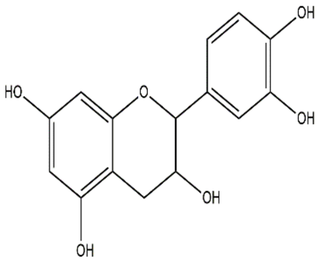	Rheum
R-2	3,4,5-Trihydroxybenzoic acid	$C_7H_6O_5$	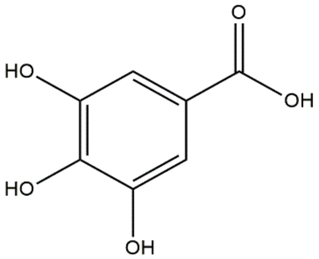	Rheum
R-3	Aloe emodin	$C_{15}H_{10}O_5$	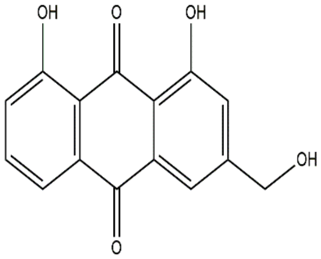	Rheum
R-4	(-)-Epicatechin-pentaacetate	$C_{25}H_{24}O_{11}$	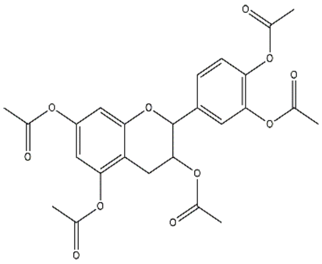	Rheum
R-5	(+)-Catechin-pentaacetate	$C_{25}H_{24}O_{11}$	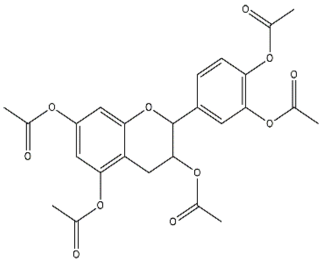	Rheum
R-6	(E)-4-Phenyl-3-Buten-2-One	$C_{10}H_{10}O$	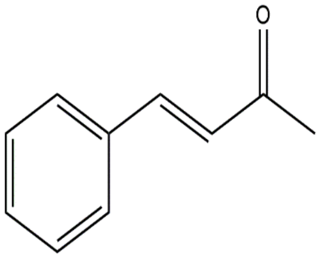	Rheum

R-7	1,8-dihydroxy-3-methoxy-2,6-dimethyl-9,10-anthraquinone	$C_{17}H_{14}O_5$	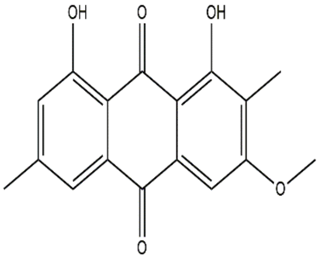	Rheum
R-8	Anthraglycoside B	$C_{21}H_{20}O_{10}$	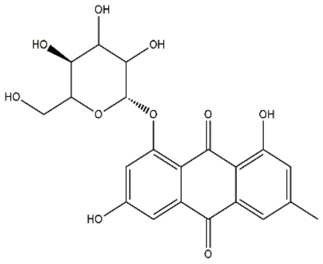	Rheum
R-9	Chrysophanol glucoside	$C_{21}H_{20}O_9$	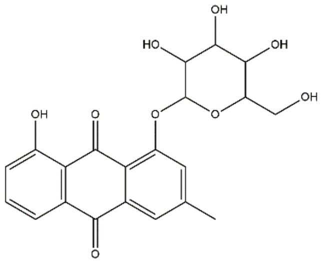	Rheum
R-10	citric acid	$C_6H_8O_7$	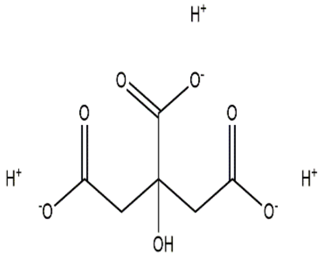	Rheum
R-11	Emodin	$C_{15}H_{10}O_5$	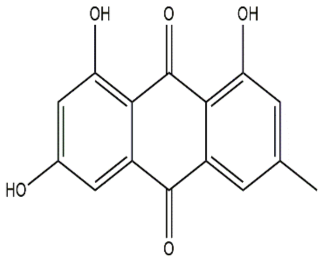	Rheum
R-12	Emodin-6-glucoside	$C_{21}H_{20}O_{10}$	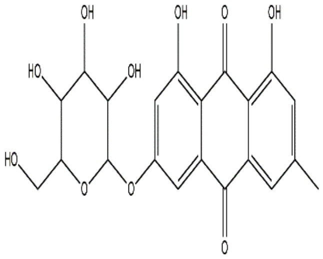	Rheum

R-13	EUPATIN	$C_{18}H_{16}O_8$	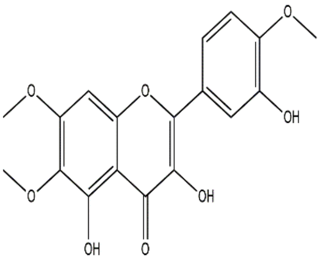	Rheum
R-14	Physcion	$C_{16}H_{12}O_5$	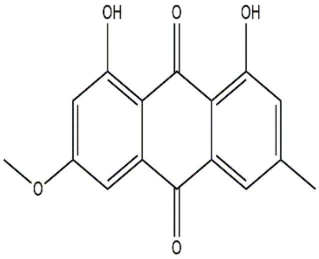	Rheum
R-15	Physcion-9-O-beta-D-glucopyranoside_qt	$C_{28}H_{32}O_{15}$	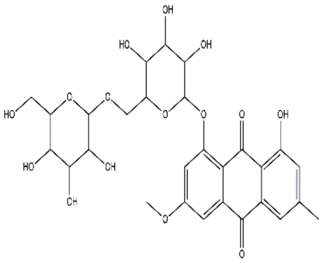	Rheum
R-16	5-acetyl-7-hydroxy-2-methyl-chromone	$C_{12}H_{10}O_4$	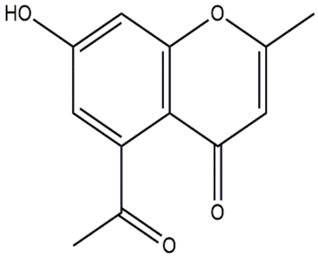	Rheum
R-17	cinnamic acid	$C_9H_8O_2$	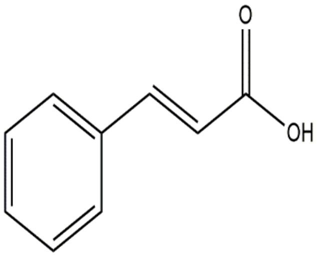	Rheum
R-18	Progesterone	$C_{21}H_{30}O_2$	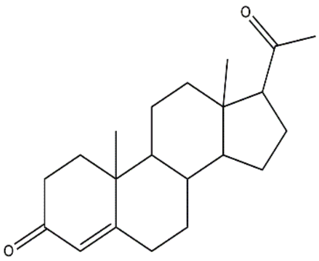	Rheum

R-19	2-Methyl Cardol	$C_{22}H_{34}O_2$	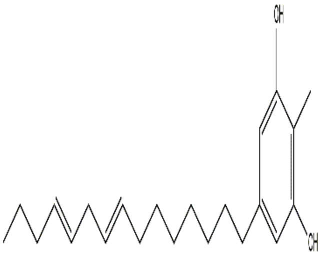	Rheum
R-20	Chrysophanol	$C_{15}H_{10}O_4$	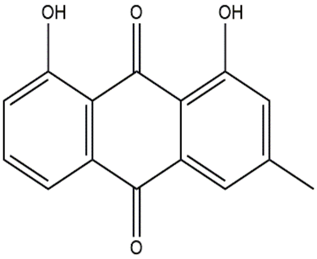	Rheum
R-21	Emodin Anthrone	$C_{15}H_{12}O_4$	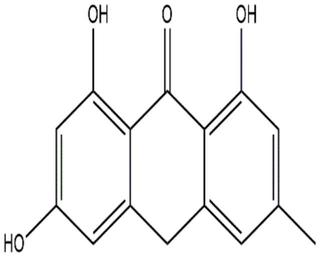	Rheum
R-22	Palmidin C	$C_{30}H_{22}O_7$	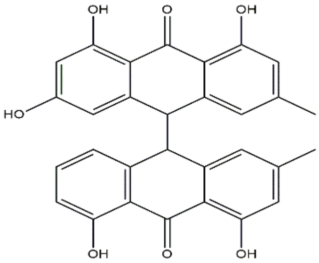	Rheum
R-23	Rhein	$C_{15}H_8O_6$	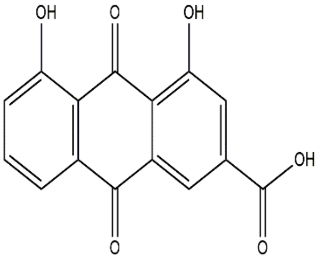	Rheum
R-24	Serotonine	$C_{12}H_{12}N_2O$	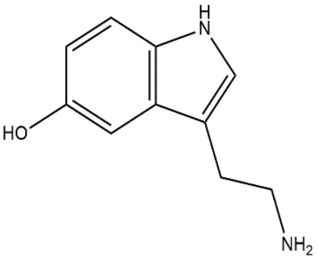	Rheum

R-25	Toralactone	$C_{15}H_{12}O_5$	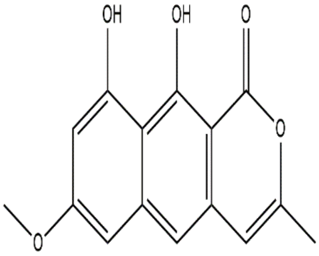	Rheum
R-26	Serotonin	$C_{10}H_{12}N_2O$	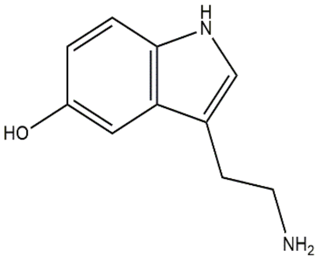	Rheum
R-27	3-Hydroxy-25-norfriedel-3,1(10)-dien-2-one-30-oic acid	$C_{29}H_{42}O_4$	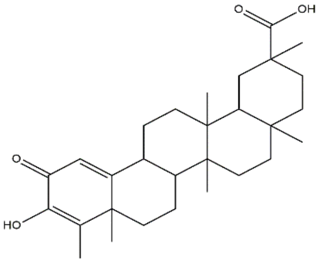	Rheum
R-28	β-sitosterol	$C_{27}H_{30}O_{12}$	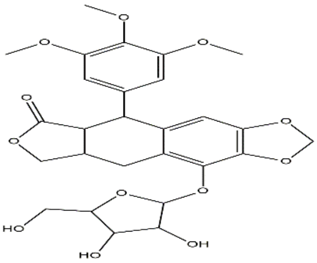	Rheum
R-29	DLA	$C_3H_6O_3$	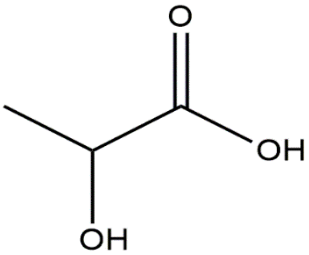	Rheum
R-30	DMR	$C_4H_6O_5$	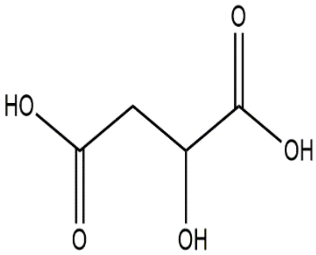	Rheum

R-31	MAE	$C_4H_4O_4$	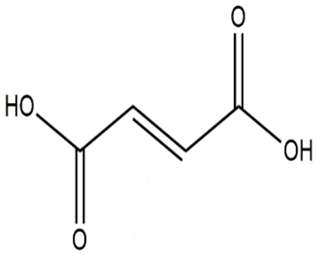	Rheum
R-32	OXL	$C_2H_2O_4$	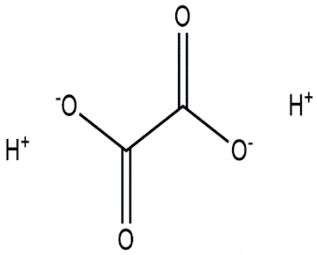	Rheum
R-33	succinic acid	$C_4H_6O_4$	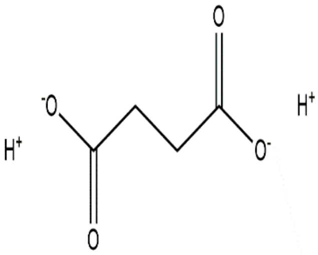	Rheum
R-34	Epicatechin	$C_{15}H_{14}O_6$	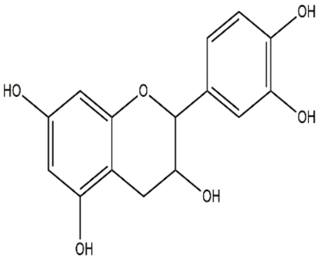	Rheum
R-35	Gallicacid	$C_7H_6O_5$	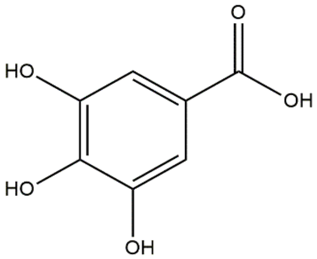	Rheum
R-36	Gallocatechin	$C_{15}H_{14}O_7$	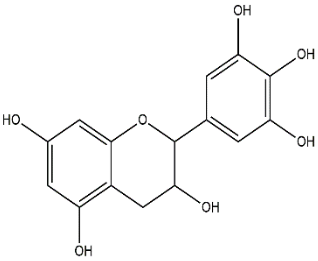	Rheum

R-37	Rheidin A	$C_{30}H_{20}O_9$	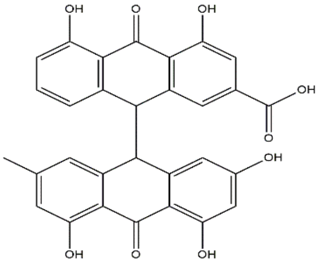	Rheum
R-38	Rheidin B	$C_{30}H_{20}O_8$	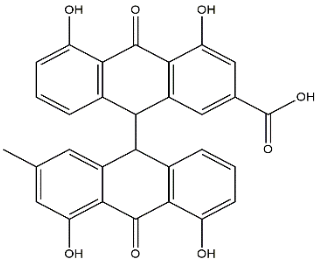	Rheum
R-39	Pontigenin	$C_{15}H_{14}O_4$	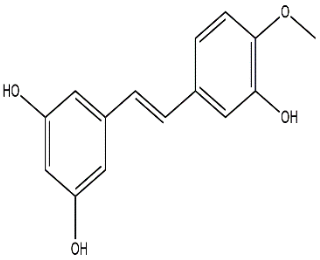	Rheum
R-40	10beta-Hydroxy-6beta-isobutyrylfuranoeremophilane	$C_{19}H_{28}O_4$	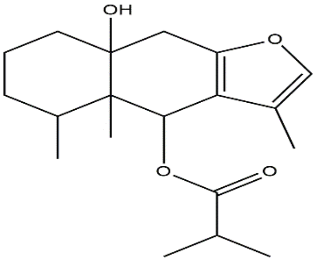	Rheum
R-41	1-O-Galloyl-glycerol	$C_{10}H_{12}O_7$	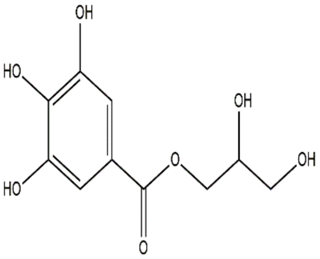	Rheum
R-42	5-Carboxy-7-hydroxy-2-methyl-benzopyran-gamma-one	$C_{11}H_8O_6$	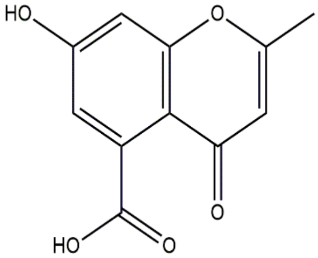	Rheum

R-43	beta-sitosterol	$C_{29}H_{50}O$	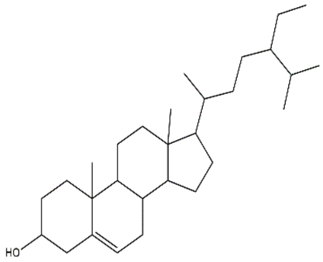	Rheum
R-44	Crysophanol	$C_{15}H_{10}O_4$	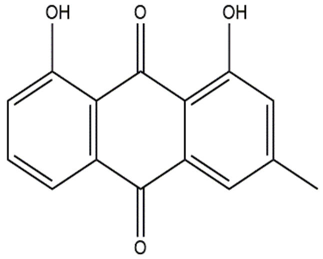	Rheum
R-45	laccaic acid D	$C_{16}H_{10}O_7$	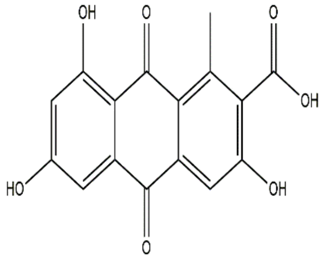	Rheum
R-46	PIT	$C_{14}H_{12}O_4$	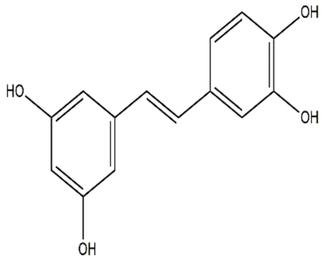	Rheum
R-47	Rhapontin	$C_{21}H_{24}O_9$	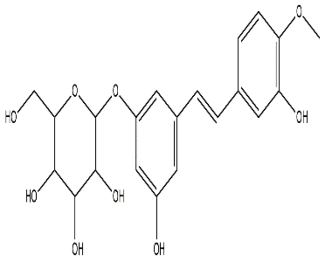	Rheum
R-48	Sennidin A	$C_{30}H_{18}O_{10}$	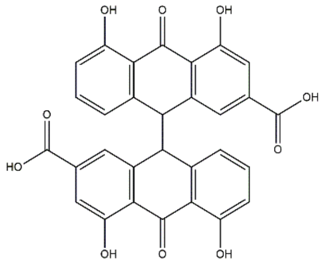	Rheum

R-49	Daucosterol_qt	$C_{29}H_{50}O$	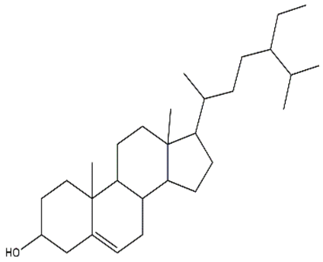	Rheum
R-50	o-Acetyltoluene	$C_9H_{10}O$	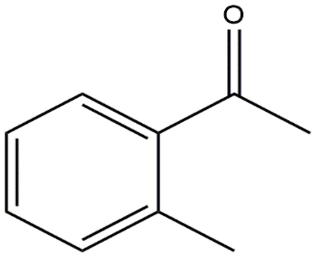	Rheum
R-51	cis-Zimtsaeure	$C_9H_8O_2$	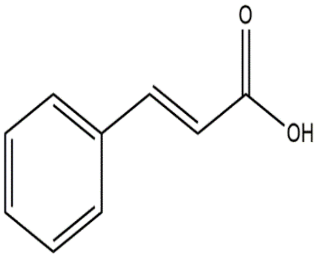	Rheum
R-52	Rottlerin	$C_{30}H_{28}O_8$	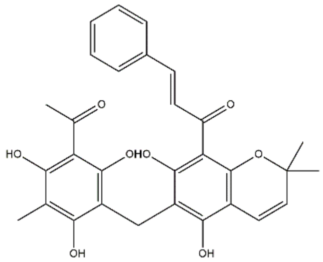	Rheum
S-1	Campesterol	$C_{28}H_{48}O$	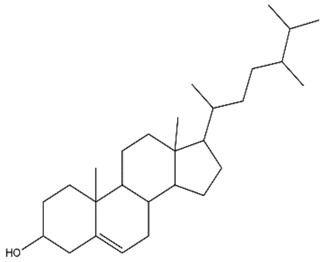	Rehmannia
S-2	Aucubin	$C_{15}H_{22}O_9$	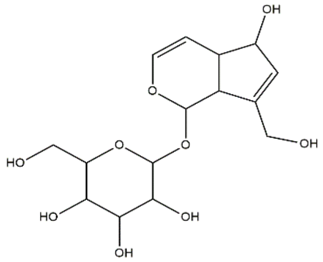	Rehmannia

S-3	Gamma-Aminobutyric Acid	$C_4H_9NO_2$	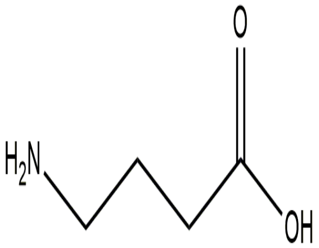	Rehmannia
S-4	Acteoside	$C_{29}H_{36}O_{15}$	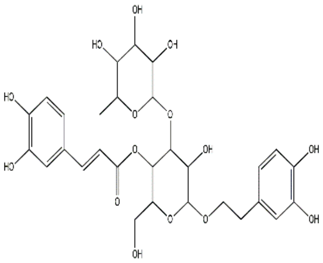	Rehmannia
S-5	Rehmannioside A	$C_{21}H_{32}O_{15}$	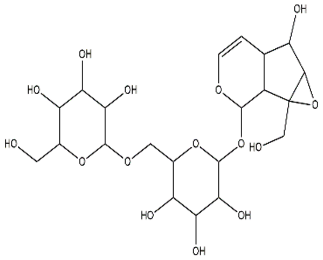	Rehmannia

Supplementary Table 2 The code and Uniprot ID of potential targets

Code	Target	Uniprot ID
T-1	FASN	P49327
T-2	F10	P00742
T-3	PROS1	P07225
T-4	PROZ	P22891
T-5	F7	P08709
T-6	F9	P00740
T-7	GGCX	P38435
T-8	VKORC1	Q9BQB6
T-9	AVP	P01185
T-10	F3	P13726
T-11	CSF1	P09603
T-12	PKD2	Q13563
T-13	SERPINF2	P08697
T-14	GAS6	Q14393
T-15	ACSL4	O60488

T-16	FGA	P02671
T-17	ESR2	Q92731
T-18	FGFR2	P21802
T-19	WNT4	P56705
T-20	TP53	P04637
T-21	NR3C2	P08235
T-22	CYP19A1	P11511
T-23	CDKN1A	P38936
T-24	CFTR	P13569
T-25	BCHE	P06276
T-26	F13A1	P00488
T-27	LDHA	P00338
T-28	KDR	P35968
T-29	NR1I2	O75469
T-30	CYP17A1	P05093
T-31	OPRK1	P41145
T-32	SRD5A1	P18405
T-33	MED12	Q93074
T-34	SMAD4	Q13485
T-35	KLF9	Q13886
T-36	FKBP4	Q02790
T-37	ESRRA	P11474
T-38	SRC	P12931
T-39	CAV1	Q03135
T-40	HSP90AA1	P07900
T-41	CEBPB	P17676
T-42	KCNN3	Q9UGI6
T-43	TRIM25	Q14258
T-44	HMGA2	P52926
T-45	NEDD4	P46934
T-46	ARID1A	O14497
T-47	TALDO1	P37837
T-48	GATA3	P23771
T-49	IL10	P22301
T-50	IDO1	P14902
T-51	SELP	P16109
T-52	GNAS	P63092
T-53	PTGER3	P43115
T-54	SERPINE1	P05121
T-55	SLC12A6	Q9UHW9
T-56	ORM1	P02763
T-57	ESR1	P03372
T-58	PTGS2	P35354
T-59	F2	P00734

T-60	LRRK2	Q5S007
T-61	PGR	P06401
T-62	VDR	P11473
T-63	AR	P10275
T-64	NR3C1	P04150
T-65	BAX	Q07812
T-66	DRD1	P21728
T-67	LYZ	P61626
T-68	PTEN	P60484
T-69	DRD2	P14416
T-70	ATP1A2	P50993
T-71	ATP2A1	O14983
T-72	P2RY12	Q9H244
T-73	MMP2	P08253
T-74	MMP9	P14780
T-75	ABCG8	Q9H221
T-76	PLG	P00747
T-77	CES1	P23141
T-78	IARS	P41252
T-79	SLC25A2	Q9BXI2
T-80	ABCC2	Q92887
T-81	F12	P00748
T-82	KLKB1	P03952
T-83	STAR	P49675
T-84	F11	P03951
T-85	KNG1	P01042
T-86	C3	P01024
T-87	STAT1	P42224

Target	Degree	Average shortest path length	Closeness centrality	Betweenness centrality

Supplementary Table 3 The topological properties of the potential targets

PTGS2	35	2.48965517	0.40166205	0.13728934
ESR1	15	2.66896552	0.374677	0.04178334
ESR2	12	2.80689655	0.35626536	0.01972041
F10	11	3.19310345	0.31317495	0.00965339
PGR	10	2.76551724	0.36159601	0.02327449
F7	7	3.24827586	0.30785563	0.00465111
VDR	6	2.84827586	0.35108959	0.00962061
NR1I2	5	3	0.33333333	0.00633892
BCHE	5	3.74482759	0.26703499	0.00083573
WNT4	5	2.95862069	0.33799534	0.00349146
FGFR2	5	2.95862069	0.33799534	0.00349146
NR3C1	5	2.84827586	0.35108959	0.01069455
TP53	4	2.94482759	0.33957845	0.00493188
FASN	4	3.68965517	0.27102804	0.00016353
DRD2	4	2.69655172	0.37084399	0.0297144
LYZ	4	3.22068966	0.31049251	0.01500104
DRD1	4	2.68275862	0.37275064	0.03131391
AR	4	2.87586207	0.34772182	0.00851301
SERPINE1	3	3.73103448	0.26802218	0.00015335
CDKN1A	3	2.95862069	0.33799534	0.00319541
CYP19A1	3	3.0137931	0.33180778	0.00243904
NR3C2	3	3.02758621	0.33029613	0.00214676
BAX	3	3.4137931	0.29292929	0.00139936
F2	3	3.04137931	0.32879819	0.02302678
OPRK1	2	3	0.33333333	0.00125424
KDR	2	3.68965517	0.27102804	0.00004002
LDHA	2	3.7862069	0.26411658	0.00004789
GAS6	2	2.82068966	0.35452323	0.01889716
F3	2	3.4137931	0.29292929	0.00062446
VKORC1	2	3.42758621	0.2917505	0.00087456
P2RY12	2	3.16551724	0.31590414	0.00682262
ATP2A1	2	2.76551724	0.36159601	0.01545134
ATP1A2	2	2.76551724	0.36159601	0.01545134
PTEN	2	2.76551724	0.36159601	0.01545134
LRRK2	2	3.06896552	0.3258427	0.01696818
ORM1	1	3.49655172	0.28599606	0
SLC12A6	1	3.49655172	0.28599606	0
PTGER3	1	3.08275862	0.32438479	0
GNAS	1	3.08275862	0.32438479	0
SELP	1	3.08275862	0.32438479	0
IDO1	1	3.08275862	0.32438479	0
IL10	1	3.08275862	0.32438479	0
GATA3	1	3.08275862	0.32438479	0
TALDO1	1	3.08275862	0.32438479	0

ARID1A	1	3.08275862	0.32438479	0
NEDD4	1	3.08275862	0.32438479	0
HMGA2	1	3.08275862	0.32438479	0
TRIM25	1	3.08275862	0.32438479	0
KCNN3	1	3.08275862	0.32438479	0
CEBPB	1	3.08275862	0.32438479	0
HSP90AA1	1	3.08275862	0.32438479	0
CAV1	1	3.08275862	0.32438479	0
SRC	1	3.08275862	0.32438479	0
ESRRA	1	3.08275862	0.32438479	0
FKBP4	1	3.08275862	0.32438479	0
KLF9	1	3.08275862	0.32438479	0
SMAD4	1	3.08275862	0.32438479	0
MED12	1	3.08275862	0.32438479	0
SRD5A1	1	3.08275862	0.32438479	0
CYP17A1	1	3.08275862	0.32438479	0
F13A1	1	3.8	0.26315789	0
CFTR	1	3.8	0.26315789	0
FGA	1	3.44137931	0.29058116	0
ACSL4	1	3.44137931	0.29058116	0
SERPINF2	1	3.44137931	0.29058116	0
PKD2	1	3.44137931	0.29058116	0
CSF1	1	3.44137931	0.29058116	0
AVP	1	3.44137931	0.29058116	0
GGCX	1	3.44137931	0.29058116	0
F9	1	3.44137931	0.29058116	0
PROZ	1	3.44137931	0.29058116	0
PROS1	1	3.44137931	0.29058116	0
STAT1	1	5.17931034	0.1930759	0
C3	1	4.02068966	0.24871355	0
KNG1	1	4.02068966	0.24871355	0
F11	1	4.02068966	0.24871355	0
STAR	1	4.02068966	0.24871355	0
KLKB1	1	4.02068966	0.24871355	0
F12	1	4.02068966	0.24871355	0
ABCC2	1	4.02068966	0.24871355	0
SLC25A2	1	4.02068966	0.24871355	0
IARS	1	4.02068966	0.24871355	0
CES1	1	4.02068966	0.24871355	0
PLG	1	4.02068966	0.24871355	0
ABCG8	1	4.13103448	0.24207012	0
MMP9	1	5.16551724	0.19359146	0
MMP2	1	5.16551724	0.19359146	0

Supplementary Table 4 The KEEG for the treatment of DUB by SDHD

Pathways	Count	P-Value	Genes	FDR
Complement and coagulation cascades	12	6.72E-11	KNG1, F12, F10, C3, SERPINF2, KLKB1, F3, F2, SERPINE1, F9, F7, PLG	8.18E-08
Pathways in cancer	15	1.78E-05	FGFR2, AR, HSP90AA1, PTGER3, PTGS2, MMP9, SMAD4,	0.021666409

			TP53, STAT1, PTEN, MMP2, WNT4, CDKN1A, BAX, GNAS	
Estrogen signaling pathway	8	3.84E-05	HSP90AA1, MMP9, FKBP4, ESR1, GNAS, ESR2, MMP2, SRC	0.046723743
Bladder cancer	5	4.88E-04	CDKN1A, MMP9, TP53, MMP2, SRC	0.59184219
Proteoglycans in cancer	9	5.01E-04	CDKN1A, CAV1, WNT4, MMP9, ESR1, TP53, MMP2, SRC, KDR	0.607468593
Hepatitis B	8	6.49E-04	CDKN1A, MMP9, BAX, SMAD4, TP53, STAT1, PTEN, SRC	0.786944169
Thyroid hormone signaling pathway	7	7.94E-04	WNT4, ESR1, TP53, MED12, ATP1A2, STAT1, SRC	0.962064641
Prostate cancer	6	0.00140601	FGFR2, AR, CDKN1A, HSP90AA1, TP53, PTEN	1.696798913
Tuberculosis	8	0.00149422	VDR, CEBPB, C3, BAX, STAT1, IL10, SRC	1.802360518
Ovarian steroidogenesis	5	0.00150096	CYP17A1, STAR, PTGS2, GNAS, CYP19A1	1.81042333
Prolactin signaling pathway	5	0.00387729	CYP17A1, ESR1, ESR2, STAT1, SRC	4.615271421
Bile secretion	5	0.00431743	ABCG8, GNAS, CFTR, ATP1A2, ABCC2	5.126637858
p53 signaling pathway	5	0.00529662	CDKN1A, BAX, SERPINE1, TP53, PTEN	6.255278434
Endocrine and other factor-regulated calcium reabsorption	4	0.00739009	VDR, ESR1, GNAS, ATP1A2	8.627033209
Staphylococcus aureus infection	4	0.01016358	SELP, C3, PLG, IL10	11.68442766
Transcriptional misregulation in cancer	6	0.01725542	CDKN1A, CEBPB, MMP9, TP53, HMGA2	19.08024849
TNF signaling pathway	5	0.02122415	CEBPB, PTGS2, MMP9, CSF1	22.96663058
Leishmaniasis	4	0.02266374	PTGS2, C3, STAT1, IL10	24.3334726
cAMP signaling pathway	6	0.04434658	DRD1, PTGER3, DRD2, GNAS, CFTR, ATP1A2	42.40418256
Neuroactive ligand-receptor interaction	7	0.04627307	DRD1, PTGER3, DRD2, OPRK1, F2, NR3C1, PLG	43.80062473
Rap1 signaling pathway	6	0.05105271	FGFR2, DRD2, CSF1, GNAS, SRC, KDR	47.13210419
Pancreatic secretion	4	0.05200314	ATP2A1, GNAS, CFTR, ATP1A2	47.77256158
Gap junction	4	0.05500464	DRD1, DRD2, GNAS, SRC	49.7486918
ABC transporters	3	0.06626564	ABCG8, CFTR, ABCC2	56.56679295
Cocaine addiction	3	0.07175697	DRD1, DRD2, GNAS	59.57367836
MicroRNAs in cancer	6	0.07243133	CDKN1A, PTGS2, MMP9, TP53, HMGA2, PTEN	59.92945834
Chagas disease (American trypanosomiasis)	4	0.0874343	C3, SERPINE1, GNAS, IL10	67.1385575
Steroid hormone biosynthesis	3	0.09205283	CYP17A1, SRD5A1, CYP19A1	69.10527352
Ubiquinone and other terpenoid-quinone biosynthesis	2	0.09398475	GGCX, VKORC1	69.89542185
PI3K-Akt signaling pathway	7	0.09943877	FGFR2, CDKN1A, HSP90AA1, CSF1, TP53, PTEN, KDR	72.0270941

Supplementary Table 5 The GO-BP pathways for the treatment of DUB by SDHD

Pathways	Genecounts	P-Value	Genes	FDR

Blood coagulation	9	2.76E-11	P2RY12, F12, F10, F3, F2, F9, F7, PLG, GAS6	4.36E-08
Transcription, DNA-templated	13	2.37E-07	PGR, VDR, AR, ESRRA, CEBPB, NR1I2, KLF9, ESR1, TP53, ESR2, NR3C1, STAT1	3.74E-04
Positive regulation of protein kinase B signaling	6	5.56E-06	F10, GATA3, F3, F7, SRC, GAS6	0.008759559
Positive regulation of sequence-specific DNA binding transcription factor activity	6	1.66E-05	CEBPB, ESR1, ESR2, PTEN, IL10	0.026139463
Negative regulation of endothelial cell apoptotic process	4	1.55E-04	GATA3, SERPINE1, GAS6, KDR	0.244768094
Positive regulation of transcription, DNA-templated	8	2.32E-04	AR, NR1I2, CEBPB, GATA3, ESR1, ESR2, STAT1	0.365841533
Positive regulation of ERK1 and ERK2 cascade	6	5.23E-04	FGFR2, SERPINF2, PTEN, SRC, GAS6, KDR	0.821623839
Uterus development	3	8.14E-04	GATA3, SMAD4, SRC	1.275397828
Fibrinolysis	3	8.14E-04	F12, F2, PLG	1.275397828
Prostate gland growth	3	0.001080713	AR, PTEN, CYP19A1	1.690236741
Positive regulation of transcription from RNA polymerase II promoter	10	0.001649139	FGFR2, VDR, AR, SERPINF2, GATA3, SMAD4, PKD2, TP53, NR3C1, IL10	2.568481604
Positive regulation of canonical Wnt signaling pathway	4	0.002342857	FGFR2, CAV1, LRRK2, SRC	3.630353644
Androgen receptor signaling pathway	3	0.003426999	AR, FKBP4, MED12	5.268162717
Positive regulation of nitric oxide biosynthetic process	3	0.003426999	HSP90AA1, ESR1, PKD2	5.268162717
Positive regulation of release of cytochrome c from mitochondria	3	0.003938083	MMP9, BAX, TP53	6.031179612
Negative regulation of interferon-gamma production	3	0.003938083	GATA3, IL10, GAS6	6.031179612
Steroid hormone mediated signaling pathway	3	0.004482296	VDR, AR, NR1I2	6.837327168
Positive regulation of intrinsic apoptotic signaling pathway	3	0.006309451	CAV1, BAX, TP53	9.496779643
Embryo implantation	3	0.007685455	MMP9, FKBP4, MMP2	11.45245789
Positive regulation of platelet-derived growth factor receptor signaling pathway	2	0.012627575	F3, F7	18.15505231
Angiotensin-activated signaling pathway involved in heart process	2	0.012627575	CAV1, SRC	18.15505231
Protein localization	3	0.015347134	CAV1, DRD2, TP53	21.63761645
Positive regulation of apoptotic process involved in mammary gland involution	2	0.018882266	VDR, BAX	25.95733298

Positive regulation of B cell apoptotic process	2	0.018882266	BAX, IL10	25.95733298
Angiogenesis	4	0.021659068	FGFR2, CAV1, SERPINE1, PTEN	29.19292369
Cell fate commitment	3	0.022839291	FGFR2, WNT4, GATA3	30.52763025
Negative regulation of protein binding	3	0.022839291	CAV1, BAX, LRRK2	30.52763025
Negative regulation of fibrinolysis	2	0.025097882	SERPINF2, SERPINE1	33.01641442
Negative regulation of cytosolic calcium ion concentration	2	0.025097882	DRD2, ATP1A2	33.01641442
Branching morphogenesis of anerve	2	0.025097882	FGFR2, DRD2	33.01641442

Supplementary Table 6 The GO-CC pathways for the treatment of DUB by SDHD

Pathways	Count	P-Value	Genes	FDR
Extracellular space	17	7.70E-06	F12, SELP, C3, MMP9, LYZ, F9, MMP2, IL10, GAS6, ORM1, WNT4, BCHE, SERPINF2, F3, KLKB1,	0.008895552

			SERPINE1, LRRK2	
Extracellular exosome	20	0.001887662	KNG1, LDHA, TALDO1, FKBP4, MMP9, LYZ, F9, CFTR, GAS6, SRC, SERPINF2, KLKB1, F3, BAX, F2, SERPINE1, PKD2, GNAS, ACSL4, LRRK2	2.158196315
Blood microparticle	4	0.004875839	KNG1, BCHE, SERPINF2, F2	5.487790868
Integral component of plasma membrane	11	0.0072771	P2RY12, DRD1, CAV1, PTGER3, DRD2, OPRK1, ATP2A1, PKD2, ATP1A2, ABCC2, KDR	8.088048025
Extracellular region	8	0.0102326	AVP, F10, KLKB1, F2, LYZ, F7, PLG, GAS6	11.19871063
Caveola	3	0.015305802	CAV1, LRRK2, SRC	16.31491026
Extracellular matrix	4	0.016226797	MMP9, F3, SERPINE1, MMP2	17.21430195
Nuclear chromatin	4	0.025535573	AR, GATA3, SMAD4, TP53	25.82185695
Apical plasma membrane	4	0.033511087	ABCG8, CFTR, ABCC2, PTEN	32.53759194
Nucleus	20	0.034103353	FGFR2, AR, ESRRA, LDHA, TALDO1, HSP90AA1, CEBPB, KLF9, ESR1, TP53, ESR2, NR3C1, HMGA2, STAT1, SRC, PGR, CDKN1A, NR1I2, GATA3	33.01343578
Nonmotile primary cilium	2	0.057688404	DRD2, PKD2	49.64843401
Endoplasmic reticulum	6	0.063252541	CAV1, BCHE, TP53, LRRK2, KDR, CYP19A1	52.97672943
Cell surface	5	0.06594799	P2RY12, SERPINF2, F3, CFTR, ABCC2	54.51568847
Axon	3	0.090272347	CYP17A1, DRD2, LRRK2	66.4624085

Supplementary Table 7 The GO-MF pathways for the treatment of DUB by SDHD

Pathways	Count	P-Value	Genes	FDR
Steroid binding	6	1.36E-07	PGR, AR, ESRRA, ESR1, ESR2, NR3C1	1.73E-04
Serine-type endopeptidase activity	9	1.16E-06	F12, F10, MMP9, KLKB1, F2, F9, F7, MMP2, PLG	0.001477441

Sequence-specific DNA binding	11	1.45E-05	PGR, VDR, AR, ESRRA, NR1I2, CEBPB, ESR1, TP53, ESR2, NR3C1	0.018417583
Steroid hormone receptor activity	5	3.85E-04	PGR, VDR, ESRRA, NR1I2, NR3C1	0.488008821
Transcription factor activity, sequence-specific DNA binding	10	0.001640547	PGR, AR, ESRRA, NR1I2, CEBPB, ESR1, TP53, ESR2, NR3C1	2.066841911
Protein heterodimerization activity	4	0.002304121	CAV1, BAX, SMAD4, TP53	2.891606423
RNA polymerase II transcription factor binding	3	0.002403823	GATA3, SMAD4, TP53	3.014973488
Transcriptional activator activity, RNA polymerase II core promoter proximal region sequence-specific binding	6	0.002579331	PGR, AR, ESRRA, GATA3, SMAD4, NR3C1	3.23178755
DNA binding	10	0.00444299	AR, CEBPB, KLF9, ESR1, TP53, ARID1A, NR3C1, HMGA2, STAT1	5.50667887
Heme binding	5	0.005774719	CYP17A1, PTGS2, IDO1, SRC, CYP19A1	7.101964618
Dopamine neurotransmitter receptor activity	2	0.013531644	DRD1, DRD2	15.91134307
Zinc ion binding	12	0.017484069	PGR, VDR, AR, ESRRA, NR1I2, GATA3, MMP9, ESR1, TRIM25, ESR2, NR3C1, MMP2	20.09770447
Steroid hormone binding	2	0.020229555	ATP1A2, NR3C1	22.89172344
Glycoprotein binding	3	0.023415255	SELP, F7, LRRK2	26.0207782
Identical protein binding	3	0.027307476	CAV1, LYZ, TP53	29.6848968
Estrogen receptor activity	2	0.033491049	ESR1, ESR2	35.16374838
ATP binding	13	0.034254176	FGFR2, HSP90AA1, FKBP4, TP53, CFTR, ATP1A2, SRC, KDR, ABCG8, IARS, ATP2A1, ABCC2, LRRK2	35.81191938
Calcium ion binding	8	0.040623172	F12, F10, F2, ATP2A1, PKD2, F9, F7, GAS6	40.99323061
RNA polymerase II core promoter proximal region sequence-specific DNA binding	5	0.043386557	PGR, AR, ESRRA, SMAD4, NR3C1	43.11904916
Cholesterol transporter activity	2	0.059484959	ABCG8, STAR	54.1635838
Dopamine binding	2	0.065874907	DRD1, DRD2	57.97088093
Chaperone binding	2	0.072222015	TP53, ATP1A2	61.46223743
Chromatin binding	5	0.073599179	AR, GATA3, SMAD4, TP53, MED12	62.18358299
